# *Arthrinium bambusicola* (Fungi, Sordariomycetes), a new species from *Schizostachyum brachycladum* in northern Thailand

**DOI:** 10.3897/BDJ.8.e58755

**Published:** 2020-12-14

**Authors:** Xia Tang, Ishani D. Goonasekara, Ruvishika S. Jayawardena, Hong Bo Jiang, Jun F. Li, Kevin D. Hyde, Ji C. Kang

**Affiliations:** 1 Engineering and Research Center for Southwest Biopharmaceutical Resource of National Education Ministry of China, Guizhou University, Guiyang 550025, Guizhou, P.R. China, Guiyang, China Engineering and Research Center for Southwest Biopharmaceutical Resource of National Education Ministry of China, Guizhou University, Guiyang 550025, Guizhou, P.R. China Guiyang China; 2 Centre of Excellence in Fungal Research, Mae Fah Luang University, Chiang Rai 57100, Thailand, Chiang Rai, Thailand Centre of Excellence in Fungal Research, Mae Fah Luang University, Chiang Rai 57100, Thailand Chiang Rai Thailand; 3 School of science, Mae Fah Luang University, Chiang Rai 57100, Thailand, Chiang Rai, Thailand School of science, Mae Fah Luang University, Chiang Rai 57100, Thailand Chiang Rai Thailand; 4 Key Laboratory for Plant Diversity and Biogeography of East Asia, Kunming Institute of Botany, Chinese Academy of Science, Kunming 650201, Yunnan, P.R. China, Kunming, China Key Laboratory for Plant Diversity and Biogeography of East Asia, Kunming Institute of Botany, Chinese Academy of Science, Kunming 650201, Yunnan, P.R. China Kunming China

**Keywords:** one new species, Bambusicolous fungi, multi-locus phylogeny, saprobic, Sordariomycetes, taxonomy

## Abstract

**Background:**

Species of the fungal genus *Arthrinium* (Sordariomycetes, Amphisphaeriales, Apiosporaceae) are often found on bamboo in Asia. They are endophytes, saprobes and important plant pathogens. The genus *Arthrinium* currently contains 92 species and is widely distributed in North and South America, Europe, Africa, Asia and Oceania.

**New information:**

In this study, a new species, *Arthrinium bambusicola* sp. nov., is described and illustrated. The new taxon is characterised by oval to broadly or irregularly round, medium brown, multi-guttulate to roughened, granular conidia, with finely pale slits in the outer edges. *Arthrinium bambusicola* can be distinguished from the closest related species *A. gutiae* by its conidial characteristics. Phylogenetic analyses of a four-locus dataset (ITS, LSU, TEF1, TUB2) confirm that *A. bambusicola* is a distinct new species.

## Introduction

The genus *Arthrinium*, with *A. caricicola* as type species, was established by Schmidt and Kunze (Kunze 1817). Species of *Arthrinium* are endophytes, saprobes and important plant pathogens of various hosts, particularly grasses and bamboo (Agut and Calvo 2004, Li et al. 2016, Dai et al. 2017, Wang et al. 2018, Jiang et al. 2019, Rashmi et al. 2019). The sexual morph is characterised by black, linear, fusiform ascostromata with a long, slit-like opening at the apex. The ascomata are globose to subglobose, with flattened bases and brown to blackish, with or without setae (Jiang et al. 2019, Pintos et al. 2019, Yang et al. 2019).

Species of *Arthrinium* produce both hyphomycetous and coelomycetous asexual morphs. The hyphomycetous morph is characterised by septate conidiophores, arising from basal cells or that are reduced to conidiogenous cells. Conidiogenous cells are holoblastic, monoblastic or polyblastic and are hyaline to pale brown, smooth or finely roughened, doliiform, ampulliform or subcylindrical and conidia are dark brown, brown to pale olivaceous and of various shapes (Hyde et al. 2016). The coelomycetous morph is immersed, black, globose to subglobose, septate, hyphoid conidiomata and hyaline to pale brown conidiophores arising from basal cells or that are reduced to conidiogenous cells. The conidiogenous cells are subhyaline to pale brown, smooth-walled or verrucose, holoblastic, monoblastic or polyblastic and cylindrical. The conidia are dark brown, smooth, globose to subglobose, with or without a germ slit or truncate scar at the base (Senanayake et al. 2015, Dai et al. 2017, Jiang et al. 2018, Yang et al. 2019). The presence of both hyphomycetous and coelomycetous asexual morphs has complicated the taxonomy of *Arthrinium* .

There are 92 species epithets for *Arthrinium* in Index Fungorum (2020). A total of 63 species have been introduced, based on the combination of morphological and molecular phylogenetic data (Wijayawardene et al. 2017, Jiang et al. 2018, Wang et al. 2018, Zhao et al. 2018, Jiang et al. 2019, Pintos et al. 2019, Yan et al. 2019, Yang et al. 2019). In this study, we propose a new species, based on morphological study and comparison with other species, in combination with phylogenetic analyses of a concatenated dataset of ITS, LSU, TEF1 and TUB2 sequences.

## Materials and methods

### Sample collection and isolation

Fresh samples of dead culms of *Schizostachyum brachycladum* (Poales, Poaceae and Bambusoideae) were collected at the campus of Mae Fah Luang University, Chiang Rai, Thailand on 7 May 2019. Single-spore isolation was performed as in Chomnunti et al. (2014). The holotype is deposited at the herbarium of Mae Fah Luang University, Chiang Rai, Thailand (MFLU) and the ex-type living culture is preserved at the Mae Fah Luang University Culture Collection (MFLUCC). Facesoffungi and Index Fungorum numbers for the new taxon were obtained (Jayasiri et al. 2015, Index Fungorum 2020).

### Morphological examination

Conidiomata present on the surface of the host were observed using a stereomicroscope (Motic SMZ-171, Wetzlar, Germany). Sections of conidiomata were taken and mounted in water on a microscope slide to observe fungal characters. Photographs were taken using a Nikon ECLIPSE Ni-U compound microscope connected with a Nikon camera series DS-Ri2. Morphological structures (conidiophores, conidiogenous cells, conidia) were measured by Image Frame Work software v. 0.9.7. Adobe Photoshop CC 2019 was used for editing the photographic plate. Colonies were described, based on the colour charts of Rayner (1970).

### DNA extraction, PCR amplification and sequencing

Genomic DNA was extracted from fresh mycelia obtained from living cultures that were grown on potato dextrose agar (PDA) for 15 days at room temperature, using the EZgene Fungal gDNA Kit (GD2416, Biomiga, San Diego, California, USA) following the manufacturer’s instructions. PCR amplification was done for the internal transcribed spacer region (ITS), the large subunit of the ribosomal RNA gene (LSU), translation elongation factor 1-alpha (TEF1) and beta-tubulin (TUB2). The following primers were used: ITS5 and ITS4 for ITS (White et al. 1990); LR0R and LR5 for LSU (Vilgalys and Hester 1990, Hopple 1994); EF1-728F and EF-2 for TEF1 (O’Donnell et al. 1998, Carbone and Kohn 1999).

PCR amplification was done in 50-μl volumes consisting of 2 μl of DNA template, 2 μl of each 10 μM forward and reverse primers, 25 μl of 2 ×Taq PCR Master Mix and 19 μl of deionised water. Cycling conditions were as follows: for ITS: initial denaturation at 94°C for 5 min, then 35 cycles of denaturation at 94°C for 45 s, annealing at 52°C for 50 s and extension at 72°C for 1 min; and final extension at 72°C for 10 min. For LSU: initial denaturation at 94°C for 5 min, then 35 cycles of denaturation at 94°C for 45 s, annealing at 52°C for 50 s and extension at 72°C for 1 min; and final extension at 72°C for 10 min. Lastly, for TEF1: initial denaturation at 94°C for 5 min; then 35 cycles of denaturation at 94°C for 1 min, annealing at 56°C for 1 min and extension at 72°C for 90 s; and final extension at 72°C for 10 min.

PCR products were checked in 1% agarose gels and sent to Sangon Biotech (Shanghai) Co. Ltd, China for sequencing, using the same primers.

### Phylogenetic analyses

Raw sequence reads were combined using BioEdit v. 7.0.5.3 (Hall 1999) and subjected to BLASTn (https://blast.ncbi.nlm.nih.gov/Blast.cgi) to find closely-related taxa. To confirm the phylogenetic position of our taxon, sequences of four loci (ITS, LSU, TEF1 and TUB2) were downloaded from NCBI GenBank (Table 1). Note that no TUB2 sequence was generated for the new species, *A. bambusicola*. Notwithstanding, this locus was included in our phylogenetic analyses to increase phylogenetic resolution. Sequences of individual loci were aligned using MAFFT v. 7 using the 'auto' option (https://mafft.cbrc.jp/alignment/server/index.html) (Katoh et al. 2019) and, where necessary, improved in BioEdit v. 7.0.5.3 (Hall 1999). Multiple loci were combined by SequenceMatrix (Vaidya et al. 2011). The alignment was trimmed using trimAl v 1.2 with the 'gappyout' option (Capella-Gutiérrez et al. 2009). A phylogenetic tree was reconstructed from the concatenated ITS–LSU–TEF1–TUB2 dataset using Maximum Likelihood (ML), Maximum Parsimony (MP) and Bayesian Inference (BI) analyses.

Phylogenetic analyses were performed using the CIPRES Science Gateway web portal (Miller et al. 2010). ML was done using the RAxML-HPC on XSEDE tool under the GTRGAMMA+I-Invar substitution model (Stamatakis et al. 2008). MP analysis was performed using the PAUP on XSEDE tool (Swofford 2002). A heuristic search with 1000 random taxa additions was used to infer MP trees. The value of MaxTrees was set to 5000, with branches of zero length collapsed and all multiple parsimonious trees saved. Parsimony score values for tree length (TL), consistency index (CI), retention index (RI) and homoplasy index (HI) were calculated for trees generated under different optimum criteria. Robustness of branches was estimated by maximum parsimony bootstrap proportions, using 100 bootstrap replicates, with tree bisection-reconnection branch swapping and a re-arrangement limit of 1000.

BI analysis was performed using the MrBayes on XSEDE tool available on the CIPRES Science Gateway (Huelsenbeck and Ronquist 2001, Miller et al. 2010, Ronquist et al. 2012). The best-fit model for each locus was selected by MrModeltest version 2.3, under the Akaike Information Criterion. Four Markov Chain Monte Carlo (MCMC) chains were run, starting from a random tree topology. The operation was stopped automatically when the average standard deviation of split frequencies fell below 0.01. Markov chains were set to run 10,000,000 generations with sampling every 1000 generations. A burn-in set at 25% was discarded. The Maximum Clade Credibility tree was inferred with the highest product of separate clade posterior probabilities (PP). Trees were visualised in FigTree version 1.4.0 and edited with Adobe Illustrator v. 51.1052.0.0 (Adobe Inc., San Jose, California, USA).

## Taxon treatments

### Arthrinium bambusicola

X. Tang, K.D. Hyde & J.C. Kang, 2020 sp. nov.

FB1F54D9-CA01-58E5-BECB-BC1495B4EE76

#### Materials

- **Type status:** Holotype. **Occurrence:** recordedBy: Xia Tang; **Taxon:** scientificName: Arthrinium bambusicola; kingdom: Fungi; phylum: Ascomycota; class: Sordariomycetes; order: Amphisphaeriales; family: Apiosporaceae; genus: Arthrinium; **Location:** country: Thailand; countryCode: TH; stateProvince: Chiang Rai; locality: Mae Fah Luang University; **Identification:** identifiedBy: Xia Tang; dateIdentified: 2019; **Event:** year: 2019; month: May; day: 7; habitat: terrestrial; fieldNotes: on dead culms of *Schizostachyum brachycladum*; **Record Level:** type: Holotype; collectionID: MFLU 20-0528; collectionCode: M19050706

#### Description

**Figure 1.**
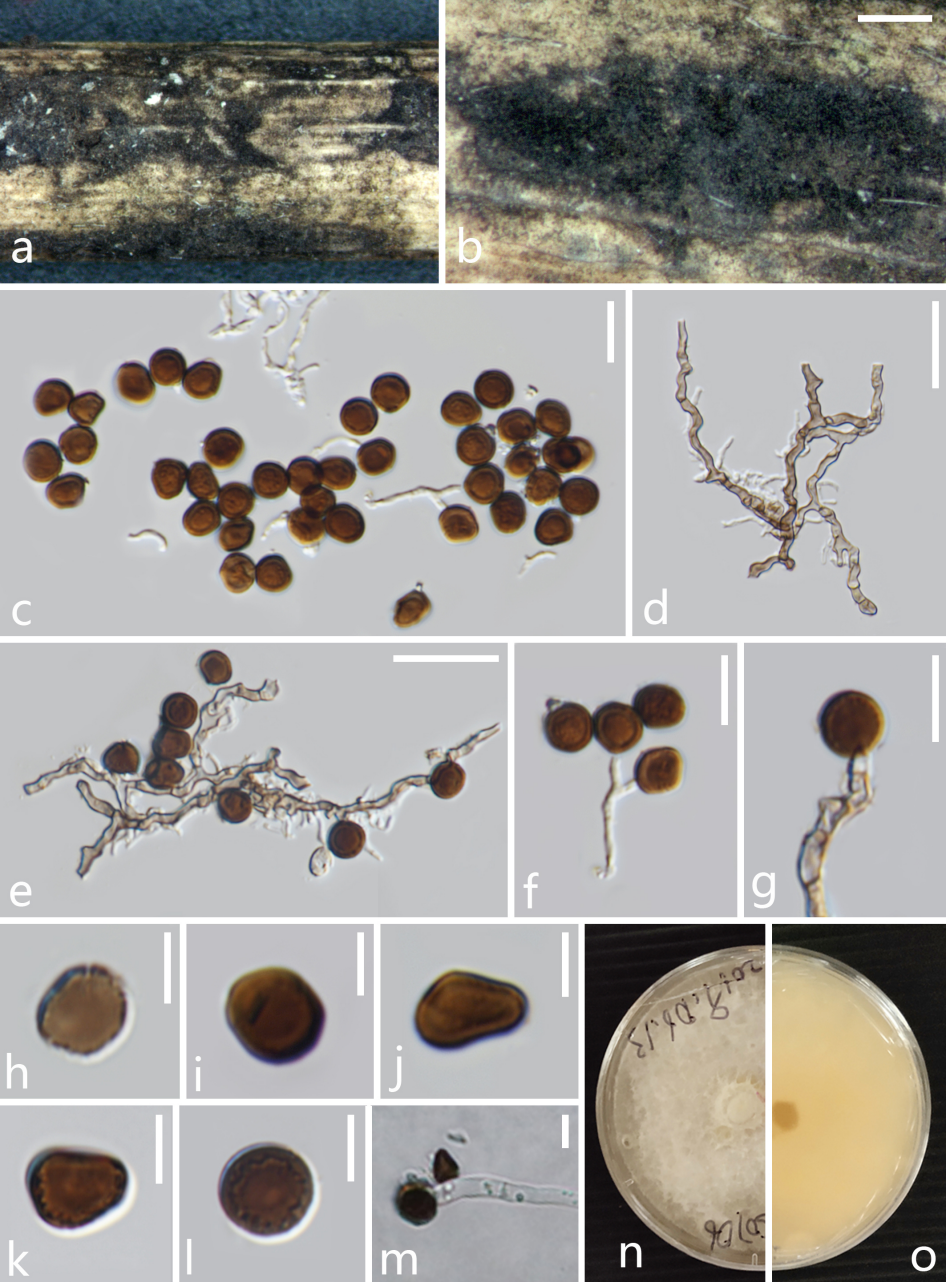
*Arthrinium bambusicola* (MFLU 20-0528, **holotype**). **a, b.** Appearance of the fungus on dead culms of *Schizostachyum brachycladum*; **c.** Conidia with mycelia; **d.** Mycelia; **e–f.** Mycelia bearing conidiogenous cells and conidia; **h–l.** Conidia; **m.** Germinated conidium; **n.** forward culture; **o.** reversed culture. Scale bars: b = 500 μm, c–e = 20 μm, f, g = 10 μm, h–m = 5 μm.

*Saprobic* on dead culms of *Schizostachyum brachycladum* (Poales, Poaceae, Bambusoideae). **Sexual morph**: Undetermined. **Asexual morph**: *Colonies* on natural substrate, superficial, gregarious, scattered, irregular, dark brown to black (Fig. 1a-b). *Mycelium* consisting of branched, septate, hyaline to dark brown (Fig. 1c-e). *Conidiophores* 0.8–3.5 μm diam. semi-micronematous to macronematous, mononematous, solitary, branched, flexuous, smooth, hyaline, aseptate when immature, becoming brown, septate when mature (Fig. 1d). *Conidiogenous cells* 1.5–4.5 × 1–4 μm, monoblastic or polyblastic, terminal, determinate, cylindrical, hyaline to light brown, smooth, aggregated, ampulliform, in clusters on aerial mycelium (Fig. 1e-g). *Conidia* pleurogenous, solitary, oval to broadly round or irregularly round, brown to medium brown, guttulate to roughened, granular, in surface view 6–8 × 6–7.8 μm (*x̅* = 6.5 × 7 μm, n = 39), in lateral view 3.5–6 × 3.5–6.5 μm (*x̅* = 4.5 × 5 μm, n = 39), with finely pale slit at outer edge (Fig. 1h-l).

##### Culture characteristics

colonies flat, spreading, with moderate, pale, aerial mycelium. On PDA, surface white, lightly yellow with patches of dirty white, reverse lightly pigmented.

##### Facesoffungi number

FoF 09162

##### Etymology

Referring to the host from which the holotype was isolated, a member of the bamboo subfamily (Bambusoideae).

##### Notes

*Arthrinium bambusicola* forms were retrieved as a sister taxon of *A. gutiae*, with relatively good support (83 ML, 77 MP, 0.99 PP). Morphologically, *A. bambusicola* differs from *A. gutiae* in having larger conidia [surface view: 5.5–8 × 6–8 μm diam., lateral view: 3.5–6 × 3.5–6.5 μm diam. versus surface view: 4.5–6 μm (*x̅* = 5.5 μm) diam., lateral view: 2–6 μm (*x̅* = 4) diam.] and irregularly rounded, guttulate to roughened conidia (*A. gutiae*: smooth-walled, globose conidia). The conidiogenous cells of *A. bambusicola* are smaller (1.5–4.5 × 1–4 μm versus 3–7× 2–4 μm). Based on pairwise nucleotide comparisons, *A. bambusicola* is different from *A. gutiae* in 31/ 620 bp (5%) of the ITS, 7/814 (0.98%) of the LSU and 44/342 bp (12%) of TEF1. Based on the combination of morphological characters and sequence data, we consider *A. bambusicola* as a distinct species.

## Analysis

### Phylogenetic analyses

**Figure 2.**
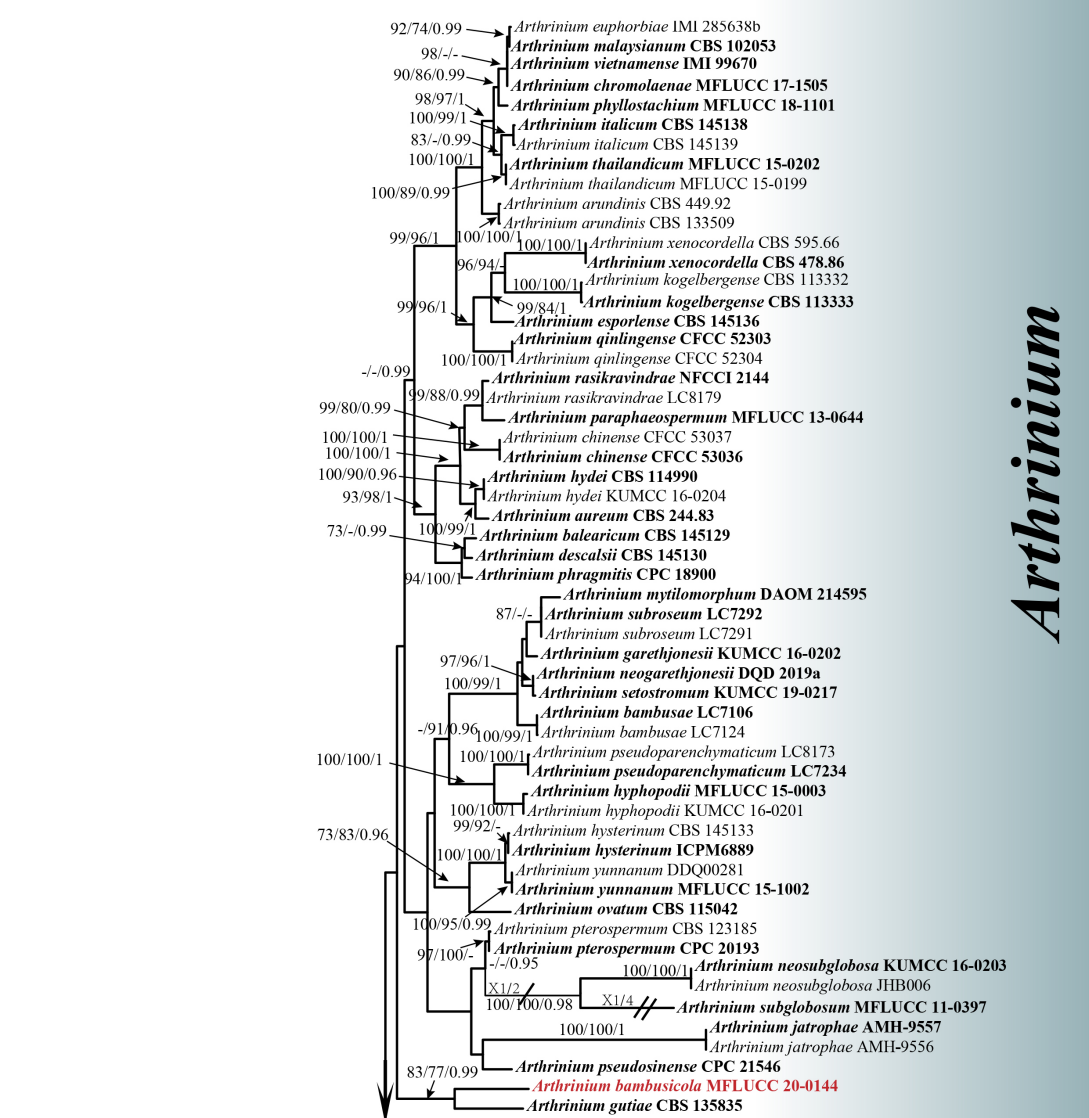
Figs 2, 3 The best-scoring RAxML tree reconstructed from a concatenated ITS–LSU–TEF1–TUB2 dataset. The tree is rooted with *Seiridium phylicae* (strains CPC 19962 and CPC 19965). ML and MP bootstrap values ≥ 70 and Bayesian PP ≥ 0.95 are shown at the nodes (ML/MP/PP). Ex-type strains are in bold; the newly-described species is highlighted in red.

**Figure 3.**
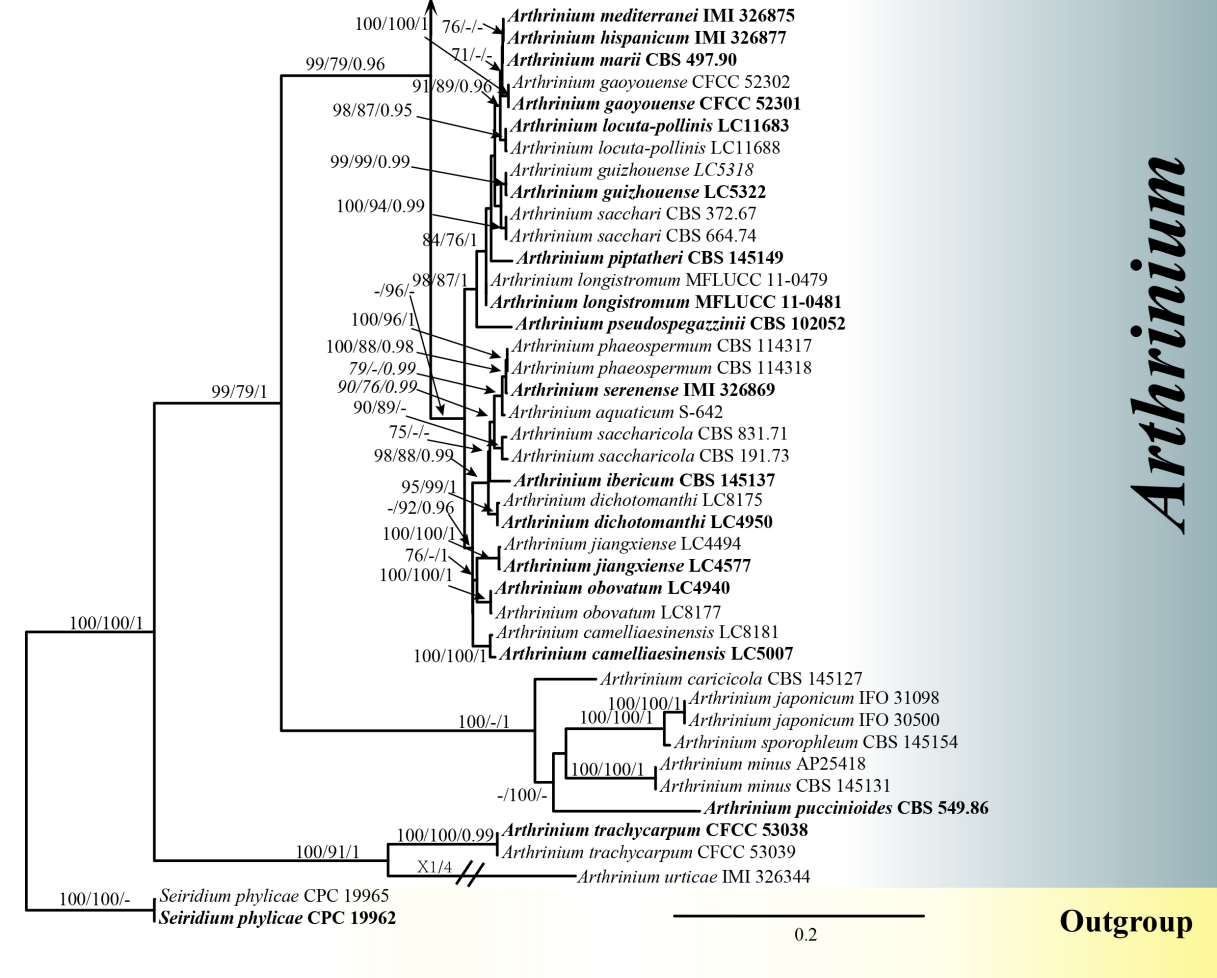
Figure 2 Continued.

The concatenated ITS–LSU–TEF1–TUB2 dataset consisted of 98 taxa with *Seiridium phylicae* (Sporocadaceae), isolates CPC 19962 and CPC 19965, as the outgroup. The data matrix consisted of 2414 total characters including gaps, of which 1126 were parsimony-informative (LSU: 1–812 bp, ITS: 813–1264 bp, TEF1: 1265–1688 bp, TUB2: 1689–2414 bp). MP (Suppl. material 3), ML (Suppl. material 4) and BI (Suppl. material 2) analyses of the concatenated dataset resulted in largely similar tree topologies. The best-scoring RaxML tree (-lnL = 25256.155227) is presented in Figs 2, 3. The most parsimonious tree showed the following values: TL = 4653, CI = 0.490, RI = 0.808, RC = 0.396 and HI = 0.510. For the Bayesian posterior probabilities analysis, the best-fit models were selected as GTR+I+G for ITS and LSU and HKY+I+G for TEF1 and TUB2; 2,895,000 generations were run. A total of 2172 trees were maintained after discarding 25% as burn-in. Bayesian PP were evaluated with a final average standard deviation of split frequencies of 0.009965.

## Discussion

The family Apiosporaceae was introduced by Hyde et al. (1998) to accommodate *Apiospora* and *Appendicospora*, based on their unique morphology. *Arthrinium* is one of the asexual morphs of *Apiospora*, along with *Cordella* and *Pteroconium* (Hyde et al. 1998). Based on molecular evidence, Crous and Groenewald (2013) confirmed that the genus *Arthrinium* belongs to Apiosporaceae (Hyde et al. 2020b, Wijayawardene et al. 2020). *Apiospora* was shown to be synonymous with *Arthrinium*, which is the oldest name (Hawksworth et al. 2011, Crous and Groenewald 2013).

*Arthrinium* species have a highly-variable morphology (Crous and Groenewald 2013, Dai et al. 2017). They produce hyphomycetous fungal structures in culture or coelomycetous fruiting bodies on their host, depending on the substrate and period of incubation (Crous and Groenewald 2013, Dai et al. 2017). However, as more species of *Arthrinium* are discovered, identification, based on morphology alone, has become very difficult because some species exhibit similar micro-morphological characters (Jiang et al. 2019).

ITS sequence data provide limited resolution to distinguish species for some *Arthrinium* species, for example, in the case of *A. phyllostachium* and *A. vietnamensis*. The ITS sequences of these species are > 99% similar. However, both species can be distinguished using the secondary barcodes TEF1 and TUB2 (Wang et al. 2018, Yang et al. 2019). In our phylogenetic analyses, *A. neogarethjonesii* (Hyde et al. 2020b) and *A. setostromum* (Jiang et al. 2019) cluster together with strong support (97 ML/96 MP/1 PP). Again, the ITS sequences of these species are > 99% similar. However, some morphological characters can be used to separate these two taxa. Whereas *A. neogarethjonesii* lacks setae, *A. setostromum* bears setae on the surface of the stromata. The former has larger stromata [1000–2000 μm × 175–250 μm versus 250–600 μm × 140–180 μm] and asci [95–125 μm × 20–25 μm versus 82.5–102.5 μm × 20–30 μm], smaller ascomata [120–230 μm × 125–230 μm versus 210–260 μm × 100–170 μm] and conidiogenous cells [10–48 μm × 4–5.5 μm versus 42–66 μm × 1.5–2.7 μm]. In their asexual morphs, *A. setostromum* has micronematous, holoblastic and monoblastic conidiogenous cells, but *A. neogarethjonesii* has basauxic conidiogenous cells. For *A. neogarethjonesii*, only ITS and LSU sequences are available; for *A. setostromum*, also a TEF1 sequence is available. Fresh collections of *A. neogarethjonesii* are necessary to generate sequences of the protein-coding genes for improved species delimitation (Hyde et al. 2020c).

*Arthrinium* species have been reported from soil debris, plants, lichens, marine algae and hive-stored pollen (Senanayake et al. 2015, Wijayawardene et al. 2017, Zhao et al. 2018), in the gut of insects (Crous et al. 2015), in nodules of human skin (Sharma et al. 2014) and especially associated with bamboo. To date, 24 *Arthrinium* species have been found in association with the bamboo subfamily Bambusoideae (Dai et al. 2016, Dai et al. 2017, Jiang et al. 2018, Wang et al. 2018, Jiang et al. 2019, Yang et al. 2019, Jiang et al. 2020, https://nt.ars-grin.gov/fungaldatabases/). *Arthrinium* species have been reported from all continents, except Antarctica (Ellis 1963, Dyko and Sutton 1979, Calvo and Guarro 1980, Von Arx 1981, Crous and Groenewald 2013, Sharma et al. 2014, Wang et al. 2018, Jiang et al. 2020).

To date, seven species of *Arthrinium* have been reported from Thailand. These are *A. bambusicola* (this study), *A. chromolaenae* (Mapook et al. 2020), *A. longistromum* (Dai et al. 2017), *A. paraphaeospermum* (Hyde et al. 2016), *A. rasikravindrii* (Dai et al. 2017), *A. subglobosum* (Senanayake et al. 2015) and *A. thailandicum* (Dai et al. 2017). Contrasting morphological features amongst these species are presented in Suppl. material 1. Six of the seven Thai species are found in association with bamboo: *Arthrinium bambusicola*, *A. longistromum*, *A. paraphaeospermum*, *A. rasikravindrii , A. subglobosum* and *A. thailandicum* (Senanayake et al. 2015, Hyde et al. 2016, Dai et al. 2017). Only *Arthrinium chromolaenae* was reported from a non-bamboo host, *Chromolaena odorata* (Asterales, Asteraceae) (Mapook et al. 2020). Further studies on this genus in Thailand and other countries, as well as from different hosts, are likely to result in the discovery of more new species (Hyde et al. 2018, Hyde et al. 2020a).

## Supplementary Material

E7F57C40-016E-5178-BE2E-D78BF526A102 10.3897/BDJ.8.e58755.suppl1 Supplementary material 1 Morphological comparison of the seven *Arthrinium* species introduced from Thailand Data type Morphological comparison Brief description The morphological comparison of seven *Arthrinium* species introduced from Thailand. File: oo_453721.txt https://binary.pensoft.net/file/453721 Xia Tang, Ishani D. Goonasekara, Ruvishika S. Jayawardena, Hong B. Jiang, Jun F. Li, Kevin D. Hyde, Ji C. Kang

DD93EC68-28BB-5213-A56D-27E5DD81ED77 10.3897/BDJ.8.e58755.suppl2 Supplementary material 2 BI output file Data type Phylogenetic output file for BI Brief description The output file of BI File: oo_478675.txt https://binary.pensoft.net/file/478675 Xia Tang

C215E929-98A3-5FF4-8C18-8A633DA10AD6 10.3897/BDJ.8.e58755.suppl3 Supplementary material 3 MP output file Data type The phylogenetic output file of MP File: oo_478682.txt https://binary.pensoft.net/file/478682 Xia Tang

235CE9A3-AD64-5680-8B6D-AF7107DB546E 10.3897/BDJ.8.e58755.suppl4 Supplementary material 4 The output file of ML Data type The phylogenetic output file of ML File: oo_478686.txt https://binary.pensoft.net/file/478686 Xia Tang

XML Treatment for Arthrinium bambusicola

## Figures and Tables

**Table 1.** Details of fungal taxa used in this study. Newly-generated sequences are indicated by ▲ after the species name; type materials are in bold.

**Species**	**Strain numbers**	**Substrates**	**Origin**	**ITS**	**LSU**	**TUB2**	**TEF 1**
*Arthrinium aquaticum*	S-642	Submerged wood	China	MK828608	MK835806	-	-
*A. arundinis*	CBS 133509	*Aspergillus flavus* sclerotium buried in sandy field	USA	KF144886	KF144930	KF144976	KF145018
*A. arundinis*	CBS 449.92	Bamboo	Canada	KF144887	KF144931	KF144977	KF145019
***A. aureum***	**CBS 244.83**	-	Japan	AB220251	KF144935	KF144981	KF145023
***A. balearicum***	**CBS 145129**	Undetermined Poaceae	Spain	MK014869	MK014836	MK017975	MK017946
***A. bambusae***	**LC7106**	Leaves of bamboo	China	KY494718	KY494794	KY705186	KY806204
*A. bambusae*	LC7124	Leaves of bamboo	China	KY494727	KY494803	KY705195	KY806206
***A. bambusicola^▲^***	**MFLUCC 20-0144**	Culms of *Schizostachyum brachycladum*	Thailand	MW173030	MW173087	-	MW183262
***A. camelliaesinensis***	**LC5007**	*Camellia sinensis*	China	KY494704	KY494780	KY705173	KY705103
*A. camelliaesinensis*	LC8181	*Brassica rapa*	China	KY494761	KY494837	KY705229	KY705157
*A. caricicola*	CBS 145127	Dead leaves of *Carex ericetorum*	China	MK014871	MK014838	MK017977	MK017948
***A. chinense***	**CFCC 53036**	*Fargesia qinlingensis*	China	MK819291	-	MK818547	MK818545
*A. chinense*	CFCC 53037	*Fargesia qinlingensis*	China	MK819292	-	MK818548	MK818546
***A. chromolaenae***	**MFLUCC 17-1505**	*Chromolaena odorata*	Thailand	MT214342	MT214436	-	MT235802
***A. descalsii***	**CBS 145130**	Dead culms of *Ampelodesmos mauritanicus*	Spain	MK014870	MK014837	MK017976	MK017947
***A. dichotomanthi***	**LC4950**	*Dichotomanthes tristaniicarpa*	China	KY494697	KY494773	KY705167	KY705096
*A. dichotomanthi*	LC8175	*Dichotomanthes tristaniicarpa*	China	KY494755	KY494831	KY705223	KY705151
***A. esporlense***	**CBS 145136**	Dead culms of *Phyllostachys aurea*	Spain	MK014878	MK014845	MK017983	MK017954
*A. euphorbiae*	IMI 285638b	*Bambusa* sp.	Bangladesh	AB220241	AB220335	AB220288	-
***A. gaoyouense***	**CFCC 52301**	Living leaves and culms of *Phragmites australis*	China	MH197124	-	MH236789	MH236793
*A. gaoyouense*	CFCC 52302	Living leaves and culms of *Phragmites australis*	China	MH197125	-	MH236790	MH236794
***A. garethjonesii***	**KUMCC 16-0202**	Dead culms of bamboo	China	KY356086	KY356091	-	-
*A. guizhouense*	LC5318	Air in karst cave	China	KY494708	KY494784	KY705177	KY705107
***A. guizhouense***	**LC5322**	Air in karst cave	China	KY494709	KY494785	KY705178	KY705108
***A. gutiae***	**CBS 135835**	Gut of a grasshopper	India	KR011352	MH877577	KR011350	KR011351
***A. hispanicum***	**IMI 326877**	Beach sand	Spain	AB220242	AB220336	AB220289	-
***A. hydei***	**CBS 114990**	Culms of *Bambusa tuldoides*	China	KF144890	KF144936	KF144982	KF145024
*A. hydei*	KUMCC 16-0204	Dead culms of bamboo	China	KY356087	KY356092	-	-
***A. hyphopodii***	**MFLUCC 15-0003**	Culms of *Bambusa tuldoides*	China	KR069110	-	-	-
*A. hyphopodii*	KUMCC 16-0201	Culms of bamboo	China	KY356088	KY356093	-	-
*A. hysterinum*	CBS 145133	*Phyllostachys aurea*	Spain	MK014875	MK014842	MK017981	MK017952
***A. hysterinum***	**ICPM6889**	Bamboo	New Zealand	MK014874	MK014841	MK017980	MK017951
***A. ibericum***	**CBS 145137**	Dead culms of *Arundo donax*	Portugal	MK014879	MK014846	MK017984	MK017955
***A. italicum***	**CBS 145138**	Dead culms of *Arundo donax*	Italy	MK014880	MK014847	MK017985	MK017956
*A. italicum*	CBS 145139	Dead culms of *Phragmites australis*	Spain	MK014881	MK014848	MK017986	-
*A. japonicum*	IFO30500	-	Japan	AB220262	AB220356	AB220309	
*A. japonicum*	IFO 31098	Leaves of *Carex despalata*	Japan	AB220264	AB220358	AB220311	-
***A. jatrophae***	**AMH-9557**	*Jatropha podagrica*	India	JQ246355	-	-	-
*A. jatrophae*	AMH-9556	*Jatropha podagrica*	India	HE981191	-	-	-
*A. jiangxiense*	LC4494	*Phyllostachys* sp.	China	KY494690	KY494766	KY705160	KY705089
***A. jiangxiense***	**LC4577**	*Maesa* sp.	China	KY494693	KY494769	KY705163	KY705092
*A. kogelbergense*	CBS 113332	Dead culms of *Cannomois virgata*	South Africa	KF144891	KF144937	KF144983	KF145025
***A. kogelbergense***	**CBS 113333**	Dead culms of Restionaceae	South Africa	KF144892	KF144938	KF144984	KF145026
*A. locuta-pollinis*	LC11688	Bee bread	China	MF939596	-	MF939623	MF939618
***A. locuta-pollinis***	**LC11683**	Hive-stored pollen of *Brassica campestris*	China	MF939595	-	MF939622	MF939616
*A. longistromum*	MFLUCC 11-0479	Dead culms of bamboo	Thailand	KU940142	KU863130	-	-
***A. longistromum***	**MFLUCC 11-0481**	Dead culms of bamboo	Thailand	KU940141	KU863129	-	-
***A. malaysianum***	**CBS 102053**	*Macaranga hullettii* stems colonised by ants	Malaysia	KF144896	KF144942	KF144988	KF145030
***A. marii***	**CBS 497.90**	Beach sands	Spain	AB220252	KF144947	KF144993	KF145035
***A. mediterranei***	**IMI 326875**	Air	Spain	AB220243	AB220337	AB220290	-
*A. minus*	AP25418	Leaves of *Carex* sp.	China	MK014872	MK014839	MK017978	MK017949
*A. minus*	CBS 145131	Dead leaves of *Carex* sp.	Germany	MK014872	MK014839	MK017978	MK017949
***A. mytilomorphum***	**DAOM 214595**	Dead blades of *Andropogon* sp.	India	KY494685	-	-	-
***A. neogarethjonesii***	**DQD 2019a**	Bamboo	China	MK070897	MK070898	-	-
*A. neosubglobosa*	JHB006	Dead culms of bamboo	China	KY356089	KY356094	-	-
***A. neosubglobosa***	**KUMCC 16-0203**	Bamboo	China	KY356090	KY356095	-	-
***A. obovatum***	**LC4940**	*Lithocarpus* sp.	China	KY494696	KY494772	KY705166	KY705095
*A. obovatum*	LC8177	*Lithocarpus* sp.	China	KY494757	KY494833	KY705225	KY705153
***A. ovatum***	**CBS 115042**	*Arundinaria hindsii*	China	KF144903	KF144950	KF144995	KF145037
***A. paraphaeospermum***	**MFLUCC 13-0644**	Dead culms of bamboo	Thailand	KX822128	KX822124	-	-
*A. phaeospermum*	CBS 114317	Leaves of *Hordeum vulgare*	Iran	KF144906	KF144953	KF144998	KF145040
*A. phaeospermum*	CBS 114318	Leaves of *Hordeum vulgare*	Iran	KF144907	KF144954	KF144999	KF145041
***A. phragmitis***	**CPC 18900**	Culms of *Phragmites australis*	Italy	KF144909	KF144956	KF145001	KF145043
***A. phyllostachium***	**MFLUCC 18-1101**	Dead culms of *Phyllostachys heteroclada*	China	MK351842	MH368077	MK291949	MK340918
***A. piptatheri***	**CBS 145149**	Dead culms of *Piptatherum miliaceum*	Spain	MK014893	MK014860	-	MK017969
***A. pseudoparenchymaticum***	**LC7234**	Leaves of bamboo	China	KY494743	KY494819	KY705211	KY705139
*A. pseudoparenchymaticum*	LC8173	Leaves of bamboo	China	KY494753	KY494829	KY705221	KY705149
***A. pseudosinense***	**CPC 21546**	Leaves of bamboo	Netherlands	KF144910	KF144957	-	KF145044
***A. pseudospegazzinii***	**CBS 102052**	*Macaranga hullettii* stem colonised by ants	Malaysia	KF144911	KF144958	KF145002	KF145045
*A. pterospermum*	CBS 123185	Leaves lesion of *Machaerina sinclairii*	New Zealand	KF144912	KF144959	KF145003	-
***A. pterospermum***	**CPC 20193**	Leaves of *Lepidosperma gladiatum*	Australia	KF144913	KF144960	KF145004	KF145046
*A. puccinioides*	CBS 549.86	Leaves of *Lepidosperma gladiatum*	Germany	AB220253	AB220347	AB220300	-
***A. qinlingense***	**CFCC 52303**	Dead culms of *Fargesia qinlingensis*	China	MH197120	-	MH236791	MH236795
*A. qinlingense*	CFCC 52304	Dead culms of *Fargesia qinlingensis*	China	MH197121	-	MH236792	MH236796
*A. rasikravindrae*	LC8179	*Brassica rapa*	China	KY494759	KY494835	KY705227	KY705155
***A. rasikravindrae***	**NFCCI 2144**	Soil	Norway	JF326454	-	-	-
*A. sacchari*	CBS 372.67	Air	-	KF144918	KF144964	KF145007	KF145049
*A. sacchari*	CBS 664.74	Soil under *Calluna vulgaris*	Netherlands	KF144919	KF144965	KF145008	KF145050
*A. saccharicola*	CBS 191.73	Air	Netherlands	KF144920	KF144966	KF145009	KF145051
*A. saccharicola*	CBS 831.71	-	Netherlands	KF144922	KF144969	KF145012	KF145054
***A. serenense***	**IMI 326869**	Food, pharmaceutical excipients, atmosphere and home dust	Spain	AB220250	AB220344	AB220297	-
***A. setostromum***	**KUMCC 19-0217**	Dead branches of bamboo	China	MN528012	MN528011	-	MN527357
*A. sporophleum*	CBS 145154	Dead leaves of *Juncus* sp.	Spain	MK014898	MK014865	MK018001	MK017973
***A. subglobosum***	**MFLUCC 11-0397**	Dead culms of bamboo	Thailand	KR069112	KR069113	-	-
*A. subroseum*	LC7291	Leaves of bamboo	China	KY494751	KY494827	KY705219	KY705147
***A. subroseum***	**LC7292**	Leaves of bamboo	China	KY494752	KY494828	KY705220	KY705148
*A. thailandicum*	MFLUCC 15-0199	Dead culms of bamboo	Thailand	KU940146	KU863134	-	-
***A. thailandicum***	**MFLUCC 15-0202**	Dead culms of bamboo	Thailand	KU940145	KU863133	-	-
***A. trachycarpum***	**CFCC 53038**	Dead branches of *Trachycarpus fortune*	China	MK301098	-	MK303394	MK303396
*A. trachycarpum*	CFCC 53039	Dead branches of *Trachycarpus fortune*	China	MK301099	-	MK303395	MK303397
*A. urticae*	IMI 326344	-	-	AB220245	AB220339	AB220292	-
***A. vietnamense***	**IMI 99670**	*Citrus sinensis*	Vietnam	KX986096	KX986111	KY019466	-
***A. xenocordella***	**CBS 478.86**	Soil from roadway	Zimbabwe	KF144925	KF144970	KF145013	KF145055
*A. xenocordella*	CBS 595.66	Soil	Austria	KF144926	KF144971	-	-
*A. yunnanum*	DDQ00281	Dead culms of *Phyllostachys nigra*	China	KU940148	KU863136	-	-
***A. yunnanum***	**MFLUCC 15-1002**	Dead culms of *Phyllostachys nigra*	China	KU940147	KU863135	-	-
***Seiridium phylicae***	**CPC 19962**	*Phylica arborea*	UK	LT853092	KC005807	LT853239	LT853189
*Seiridium phylicae*	CPC 19965	*Phylica arborea*	UK	LT853093	KC005809	LT853240	LT853190

**Notes**: Newly-generated sequences are indicated by ▲ after the species name. Ex-type strains are in bold. Abbreviations: **AMH**: Ajrekar Mycological Herbarium, Pune, Maharashtra, India; **CBS**: Westerdijk Fungal Biodiversity Institute, Utrecht, Netherlands; **CFCC**: China Forestry Culture Collection Center, Beijing, China; **CPC**: Culture collection of Pedro Crous, housed at the Westerdijk Fungal Biodiversity Institute; **DAOM**: Canadian Collection of Fungal Cultures, Ottawa, Canada; **DDQ**: D.Q. Dai; **ICMP**: International Collection of Microorganisms from Plants, New Zealand; **IFO**: Institute for Fermentation, Osaka, Japan; **IMI**: Culture collection of CABI Europe UK Centre, Egham, UK; **JHB**: H.B. Jiang; **KUMCC**: Culture collection of Kunming Institute of Botany, Yunnan, China; **LC**: personal culture collection of Lei Cai, housed in the Institute of Microbiology, Chinese Academy of Sciences, China; **MFLUCC**: Mae Fah Luang University Culture Collection, Chiang Rai, Thailand; **NFCCI**: National Fungal Culture Collection of India.
